# Dietary Patterns Prior to Pregnancy and Associations with Pregnancy Complications

**DOI:** 10.3390/nu10070914

**Published:** 2018-07-17

**Authors:** Megan Jarman, Nonsikelelo Mathe, Fatemeh Ramazani, Mohammadreza Pakseresht, Paula J. Robson, Steven T. Johnson, Rhonda C. Bell, the APrON and ENRICH study teams

**Affiliations:** 1 Li Ka Shing Centre for Health Research Innovation, Department of Agricultural, Food and Nutritional Sciences, Division of Human Nutrition, University of Alberta, Edmonton, AB T6G 2E1, Canada; jarman@ualberta.ca (M.J.); pakseres@ualberta.ca (M.P.); 2 Alliance for Health Outcomes Research in Diabetes (ACHORD), University of Alberta, Edmonton, AB T6G 2E1, Canada; mathe@ualberta.ca (N.M.); ramazani@ualberta.ca (F.R.); sjohnson@athabasca.ca (S.T.J.); 3 Cancer Research, CancerControl Alberta, Alberta Health Services, Edmonton, AB T5J 3H1, Canada; Paula.Robson@albertahealthservices.ca; 4 Faculty of Health Disciplines, Athabasca University, Athabasca, AB T9S 3A3, Canada

**Keywords:** pre-pregnancy, dietary patterns, principal components analysis, gestational complications

## Abstract

Few studies have explored pre-pregnancy diet and its relationship with pregnancy outcomes. The objectives of this study were to: (1) derive pre-pregnancy dietary patterns for women enrolled in a prospective cohort in the province of Alberta, Canada; (2) describe associations between dietary patterns and socio-demographic characteristics; and (3) describe associations between dietary patterns and pregnancy complications. Upon enrolment into the Alberta Pregnancy Outcomes and Nutrition (APrON) study (median age of gestation, 17 weeks), women (*n* = 1545) completed a validated 142-item food frequency questionnaire recording food and beverages consumed “in the 12 months prior to pregnancy”. Other assessments included pre-pregnancy body mass index (BMI), gestational weight gain, gestational hypertension, gestational diabetes, and socio-demographic characteristics. Dietary patterns were derived using principal components analysis. Scores were calculated to represent adherence with each dietary pattern retained. Four dietary patterns were retained, accounting for 22.9% of the variation in the overall diet. Dietary patterns were named the “healthy”, “meat and refined carbohydrate”, “beans, cheese and salad” or “tea and coffee” patterns. Higher “healthy” pattern scores prior to pregnancy were associated with lower odds of developing gestational hypertension during pregnancy (adjusted Odds Ratio (OR): 0.6, 95% Confidence Intervals (CI): 0.4, 0.9). Diet prior to pregnancy is an important target for interventions and may reduce the likelihood of developing complications such as gestational hypertension during pregnancy.

## 1. Introduction

Diet prior to and during pregnancy may have implications for the health of both mother and baby. Studies of the developmental origins of health and disease have shown that the nutritional environment that mothers provide during pregnancy is important for the optimal health, development, and long term chronic disease risk of the baby [1,2]. In women, data from prospective cohort studies has indicated that during pregnancy, consuming a diet rich in fruits, vegetables, lean meat/fish, and wholegrains while being low in energy dense–nutritionally poor food is associated with a lower risk of exceeding the Institute of Medicine (IOM) gestational weight gain (GWG) guidelines [3], and lower risk of developing complications such as gestational hypertension [4] or gestational diabetes [5,6,7]. In 2016, the Society for Obstetricians and Gynaecology Canada released a report on the consensus on female nutrition across the life-course in order to achieve optimal health for both women and their offspring [8]. The report recommended that all women consume a balanced and varied diet consisting of frequent intakes of vegetables, fruit, whole-grains, low-fat diary, lean meat and fish, and legumes and nuts, with lower consumption of red and processed meat and sugar-sweetened beverages [8]. During pregnancy women, are recommended to also increase calorie intakes by around 300 kcal/day from the second trimester but to do so by consuming extra portions of vegetables and fruit, whole-grains, low-fat dairy or alternatives, or lean meat/fish or alternatives, as opposed to increasing consumption of energy-dense, nutrient poor foods [9]. A diet in line with these recommendations aims to meet a woman’s own nutritional requirements as well as facilitate healthy development of her foetus throughout the pregnancy [8]. 

Diet during pregnancy has therefore been a focus for a number of interventions to improve pregnancy health outcomes. Despite large-scale interventional Randomized Controlled Trials (RCT) such as the LIMIT trial in Australia [10] and the UK Pregnancies Better Eating and Activity Trial (UPBEAT) in the UK [11] being successful in improving dietary behaviours of women during pregnancy, there were no significant effects on reducing pregnancy complications such as gestational diabetes and hypertension. Therefore there has recently been an increasing focus on the influence of preconception health on pregnancy outcomes [12]. There is evidence to suggest that patterns of diet are relatively stable [13,14] and have been shown to track from prior to during pregnancy [15]. Therefore, it is likely that establishing healthy eating behaviours prior to becoming pregnant could be a key target for intervention to improve pregnancy health. There is some evidence that patterns of diet prior to pregnancy which include higher intakes of red and processed meat and low intakes of fruits, vegetables, and whole-grains are associated with an increased risk of developing gestational diabetes [16,17,18] or gestational hypertension [19]. However, these findings are based on data from two cohort studies (one in the USA and one in Australia), which were collected 15–25 years ago and it is possible that patterns of food choice have changed over time. Therefore, more contemporary diet data would be advantageous. In addition, these studies only included gestational hypertension or gestational diabetes as outcome measures and neither assessed associations between dietary patterns and excess or inadequate gestational weight gain. Furthermore, both research groups concluded that assessment of dietary patterns prior to pregnancy and associations with pregnancy complications needed to be carried out in other prospective cohort studies in order to expand the evidence base.

Diet has often been examined by relationships between single nutrients or foods, e.g., fat, carbohydrate, or red meat, and a particular state (e.g., disease or weight gain). Whilst this is useful for hypothesizing the mechanism for the diet–disease relationship it does not account for the complexity of human dietary behaviours where foods, and therefore nutrients, are often consumed in combination. Dietary pattern analysis detects groups of foods that tend to cluster (i.e., consumed together) and their combined effect on the risk for diseases or their effect in disease progression can be determined [20].

Therefore this study aims to: (1) derive pre-pregnancy dietary patterns using principal component analysis for women enrolled in a prospective cohort study (Alberta Pregnancy Outcomes and Nutrition; APrON) in the province of Alberta, Canada; (2) describe associations between dietary patterns and socio-demographic characteristics; and (3) describe associations between dietary patterns and pregnancy complications (including gestational diabetes, excessive gestational weight gain, and gestational hypertension).

## 2. Methods

### 2.1. Study Population and Setting

Data were obtained from women who participated in the prospective Alberta Pregnancy Outcomes and Nutrition (APrON) cohort [21]. Briefly, APrON is an ongoing prospective cohort study of 2189 women in pregnancy and their children in the metropolitan areas of Alberta, Canada. APrON’s primary aims were to determine the relationships between maternal nutrient intake and status, before, during, and after gestation and maternal mood, birth and obstetric outcomes, and neurodevelopment in children [21]. Women were recruited between May 2009 and November 2012 and were eligible for the study if they were >16 years of age, at <27 weeks of gestation, and literate in English. The Health Research Ethics Boards at the University of Alberta and the University of Calgary granted study approval and participants gave written informed consent prior to completing any assessments. 

### 2.2. Dietary Assessment

Diet prior to pregnancy was assessed by a Food Frequency Questionnaire (FFQ), based on the National Cancer Institute’s Diet History Questionnaire for Canadians [22], and has been validated for assessing pre-pregnancy diet [23]. The FFQ was completed by participants during their first study visit. The FFQ contained 154 questions covering 142 different food and drink items. Participants were asked to report their frequency of consumption and portion sizes of these food and drinks in the 12 months prior to becoming pregnant. Frequency options were “never”; “1–6 times per year”; “7–11 times per year”, “once a month”; “2–3 times per month”; “once a week”; “2 times a week”; “3–4 times per week”; “5–6 times per week”, “once a day”; and “2 or more times a day”. Data were converted into daily frequencies of consumption following data entry. Daily calorie intake was derived by entering portion size data for each food and drink item into FoodProcessor version 10.14 [24] which linked data from the questionnaire to the Canadian Nutrient File. Once calories per portion was determined, this was multiplied by each participant’s daily frequency of consumption for the corresponding food and drink item and summed. Those with unlikely daily energy intakes of <600 kcal or >3500 kcal [25] per day were excluded from the analysis (*n* = 53)

### 2.3. Socio-Demographic and Anthropometric Assessments

Socio-demographic characteristics and behaviours including age, education, household income, marital status, occupation, and smoking history were assessed by questionnaire at each participant’s first study visit. Women reported their weight prior to pregnancy. At the first study visit trained researchers measured women’s height to the nearest 0.1 cm using a Charder HM200P Portstad portable Stadiometer (manufactured in Taiwan) and also women’s weight to the nearest 0.01 kg using a Healthometer Professional 752KL (manufactured in McCook, Chicago, IL, USA). Women were weighed at each subsequent study visit a maximum of three times during pregnancy. At the three-month postpartum visit women reported their highest weight in the pregnancy. Self-reported weight prior to pregnancy and measured height were used to calculate pre-pregnancy body mass index (BMI) in kilograms/metre^2^ (kg/m^2^). Total gestational weight gain was calculated by subtracting pre-pregnancy weight from the highest weight in pregnancy. Further details on the calculations and validity of pre-pregnancy BMI and GWG in this cohort have been published previously [26].

### 2.4. Pregnancy Complications

Incidence of gestational hypertension (early diagnosis < 24 weeks or later diagnosis ≥ 24 weeks) and gestational diabetes was assessed from the birth record. Women recruited to the APrON study provided their Alberta Health personal health number and consented to access to their health records during pregnancy and delivery. 

### 2.5. Physical Activity

Physical activity prior to pregnancy was assessed using the Baecke Questionnaire [27,28]. The questionnaire was modified to refer to physical activity prior to pregnancy. The questionnaire consists of three sections which asked participants to report the intensity and frequency of physical activity that they accrued over the month prior to becoming pregnant while: (1) at work, (2) playing sports, and (3) doing leisure-time activities. Physical activity from each of these sources was scored between 1 and 5 and a total physical activity score was calculated as a summation of all three scores resulting in a score between 5 (low activity) and 15 (high activity).

### 2.6. Dietary Patterns Analysis

**Figure 1:**
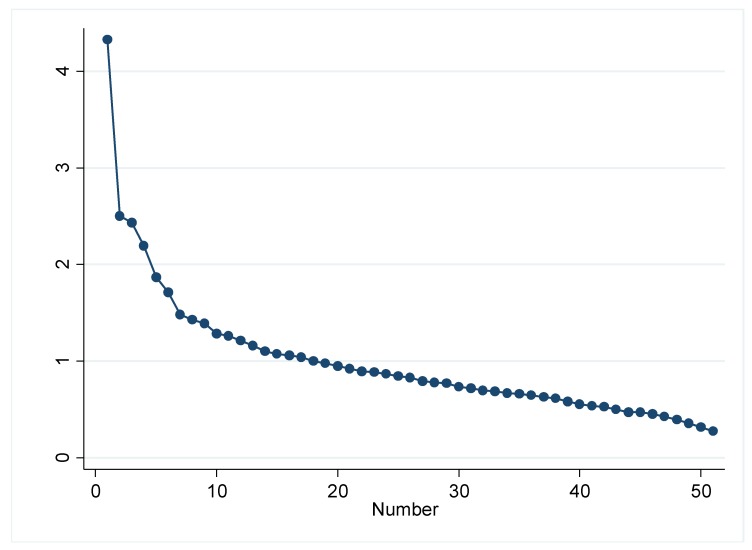
Scree plot of eigenvalues from principal component analysis (PCA) following orthogonal rotation. Scree plot indicating variance explained by number of principal components retained. Components with an eigenvalue > 2 were retained.

Principal component analysis (PCA) was conducted on dietary data derived from the FFQ. The 142 food items from the FFQ were collapsed into 51 food groups according to similarity of food type and nutritional composition in a similar process to other dietary pattern studies [29]; the food groups and the items within them are shown in supplementary Table S1. The daily frequency of consumption of foods in each food group was summed. PCA was run on the correlation matrix of the frequency of consumption of the 51 food groups following which orthogonal varimax rotation was applied [30]. The number of components to be retained were considered at the point at which the scree plot (Figure 1) levelled off (i.e., eigenvalue of greater than two) [31]. Food groups with coefficients ≥ 0.20 were used to describe the component and provide the dietary pattern interpretation. The coefficients represent covariance between the foods and the overall component. Foods with positive coefficients were positively associated with the component, whilst the opposite was true for negative coefficients. Scores were calculated to represent women’s adherence with each component retained. Scores were derived by multiplying the frequency of consumption of each food group by its corresponding coefficient for the component and then summing. These dietary pattern scores were then standardized and expressed as *z*-scores [32].

### 2.7. Statistical Analysis 

Associations between socio-demographic variables (age, education, family income, marital status, smoking history, and physical activity) and dietary pattern scores were assessed in multivariate linear regression models. Subsequently, associations between dietary pattern scores and pregnancy complications (gestational diabetes, excessive gestational weight gain and gestational hypertension) were assessed in forward step-wise logistic regression models. Covariates and confounders were included; those variables with a univariate association of *p* ≤ 0.2, and women’s age in years, were included in the final multivariate analyses. Those participants with a pre-pregnancy BMI categorized as underweight were excluded from the final analyses due to small numbers (*n* = 48). All analyses were also adjusted for total energy intake (kcal/day). Statistical analysis was conducted using STATA SE 14.0, Statacorp (College Station, TX, USA) [33]. All significant differences or associations were based at *p* < 0.05 level.

## 3. Results

A total of 1598 participants completed the FFQ upon enrolment to the study (median gestational age at recruitment was 17 weeks) of which 53 were excluded for implausible energy intakes leaving a final sample for analysis of 1545. Participants were on average 31.4 (±4.2) years old. The majority (72%) were university educated, had an annual household income of ≥CAD$100,000 (58%), were married or cohabiting (97%), and self-identified as Caucasian (84%). Prior to pregnancy, most of the women (65%) had a normal BMI, with a third of women (33%) considered overweight or obese. During pregnancy, 48% gained weight in excess of the IOM gestational weight gain guidelines, and very few smoked (1%). In total 93 (6%) women developed gestational hypertension and 49 (3%) developed gestational diabetes (Table 1).

### 3.1. Dietary Patterns

Four interpretable components were retained and examined as dietary patterns. These patterns cumulatively explained 22.9% of the total variance in diet. The dietary patterns, food groups and coefficients are described in Table 2. The first dietary pattern explained 7% of the variance in diet and was characterized by frequent intakes of other vegetables, green vegetables, fruit (excluding juice), orange vegetables, oils, brown pasta or rice, fish, tomatoes, and white pasta. Pattern one was therefore named the “healthy pattern”. The second dietary pattern explained 5.9% of the variance in the diet, and was characterized by more frequent intakes of red meat, processed meat, fries and roast potatoes, white bread, and boiled potatoes. Pattern 2 was therefore named the “meat and refined carbohydrates pattern”. The third dietary pattern explained 5.6% of the variance in diet, and was characterized by frequent intakes of beans and pulses, cheese, and salad vegetables and was therefore named the “beans, cheese and salad” pattern. The fourth dietary pattern explained 4.4% of the variance in diet and was characterized by more frequent intakes of coffee, tea (both regular and decaffeinated), reduced-fat milk, full-fat milk, cream and added sugar. Therefore, the final dietary pattern was named the “tea and coffee pattern”.

### 3.2. Socio-Demographic Associations with Patterns

Associations between socio-demographics and dietary pattern scores from multivariate regression models are displayed in Table 3. Those with higher levels of education (β 0.27 *p* ≤ 0.001), from a non-Caucasian ethnicity (β 0.52 *p* ≤ 0.001) and who reported being more physically active prior to pregnancy (β 0.04 *p* = 0.049) tended to have higher “healthy” pattern scores. In addition, those who were obese prior to pregnancy had lower “healthy” pattern scores (β −0.23 *p* = 0.012) compared to those who were of normal weight. Women with higher levels of education (β −0.13 *p* = 0.008) had lower “meat and refined carbohydrate” pattern scores and those from a non-Caucasian ethnicity (β 0.38 *p* ≤ 0.001) tended to have higher “meat and refined carbohydrate” pattern scores. The “bean, cheese and salad” pattern score was positively associated with higher household income (β 0.16 *p* = 0.012), and having a higher total physical activity score (β 0.05 *p* = 0.02) prior to pregnancy but negatively associated with being of non-Caucasian ethnicity (β −0.43 *p* ≤ 0.001). Finally, those who were older (β 0.02 *p* = 0.03), tended to have higher “tea and coffee” pattern scores.

### 3.3. Associations between Dietary Pattern and Pregnancy Complications

Associations between dietary pattern scores and gestational hypertension were first explored in univariate analyses. This showed that there was a univariate association between the healthy pattern score and lower odds of developing gestational hypertension (Odds Ratio (OR): 0.7 95% Confidence Interval (CI) 0.6, 0.9).The association between the “healthy” pattern score and gestational hypertension remained significant in a multivariate model adjusting for covariates. Table 4 shows the results from the forward step-wise logistic regression model with gestational hypertension as the outcome. This shows that women with in a higher BMI category prior to pregnancy and those with higher household incomes had higher odds of developing gestational hypertension, whereas those with higher “healthy” pattern scores had lower odds, such that for each standard deviation increase in “healthy” pattern score odds of developing gestational hypertension decreased by 0.6 (95% CI: 0.4, 0.9). There were no associations between any other dietary pattern score and gestational hypertension

Univariate associations between dietary pattern scores and gestational diabetes were not observed. However there was an association between higher levels of physical activity prior to pregnancy and lower odds of developing gestational diabetes (OR 0.8 95% CI: 0.6, 0.9) which remained significant in an adjusted analysis. In a multivariate model those in a higher BMI category prior to pregnancy (OR 2.2 95% CI: 1.4, 3.5) or who were older (OR 1.2 95% CI: 1.1, 1.3) had higher odds of developing gestational diabetes. Whereas those who had a higher total physical activity score prior to pregnancy tended to have lower odds of developing gestational diabetes (OR 0.7 95% CI: 0.5, 0.9) (supplemental Table S2). This analysis was additionally adjusted for gestational weight gain, parity, education, ethnicity and total daily energy intake.

Finally, in a univariate analysis those with a higher “tea and coffee” pattern score were more likely to exceed GWG guidelines (1.2 95% CI: 1.0, 1.4), however this association was attenuated and become non-significant following adjustment for pre-pregnancy BMI and level of education (OR 1.1 95% CI: 0.9, 1.3) (supplemental Table S3). 

## 4. Discussion

In this study, dietary patterns prior to pregnancy were examined and associations with pregnancy complications assessed. Four interpretable dietary patterns were derived which explained a total of 22.9% of the variance in total diet. These patterns were named “healthy”, “meat and refined carbohydrates”, “beans, cheese and salad” and “tea and coffee”, reflecting the food groups that characterized them. In total, 6% of women in the APrON cohort developed gestational hypertension, which was slightly higher than the average incidence for Alberta reported in 2011, which was 5.1% [34]. In contrast, 3% of women in the APrON cohort developed gestational diabetes, which was slightly lower than the average incidence for Alberta reported in 2011 of 4.4% [35]. Consuming a “healthy” pattern of diet, characterized by frequent intakes of vegetables, fruit, oils, fish, and pasta/rice prior to pregnancy, was associated with lower odds of developing gestational hypertension, independent of confounders such as pre-pregnancy BMI, gestational weight gain and socio-demographic characteristics. In an adjusted analysis, a higher overall pre-pregnancy physical activity was associated with lower odds of developing gestational diabetes.

The dietary patterns identified in this study are consistent with those defined among other western populations. The most common dietary pattern described previously include a healthy pattern, sometimes referred to as “prudent” which is often characterized by high fruit and vegetable intake, whole grains, and lean meats [36]. Two large studies of dietary patterns prior to pregnancy also described patterns similar to our “healthy” pattern as the first component [18,37]. The Southampton Women’s Survey (SWS) assessed dietary patterns in >6000 women recruited as non-pregnant women of childbearing age [37]. The main dietary pattern they described was the “prudent diet”. The prudent diet was similar to the “healthy” pattern reported here, being reflective of frequent fruit, vegetable, and rice/pasta intakes, however it was additionally characterized by lower intakes of fries, crisps, and confectionery [37]. Additionally dietary patterns analysis of 13,110 women prior to pregnancy in the Nurses’ Health Study II (NHSII) described a “prudent” diet which also reflected higher intakes of fruit, green vegetables, fish and poultry [38]. Our study advances the evidence base by utilizing more contemporary data and in the Canadian context. Of note is the similarity between the prudent pre-pregnancy dietary pattern identified in these cohorts assessed >20 years ago in different Western countries and the “healthy” pattern found in our study, highlighting the consistency of this pattern, which explains most of the variation in the overall diet of women of childbearing age.

The “meat and refined carbohydrate” pattern we described is similar to the second component from the Nurses’ Health study II in that it was reflective of higher intakes of processed meat, red meat, refined grains and fries but in addition to sweets and pizza [18]. The “beans, cheese and salad” pattern may be reflective of a vegetarian pattern with protein sources coming from the beans and pulses and cheese. To our knowledge, the only similar pattern reported in the literature was from the Avon Longitudinal Study of Parents and Children (ALSPAC), which also described a vegetarian pattern in women; however this was additionally negatively correlated with meat consumption, which was not the case in our study [38]. It is possible that a dietary pattern of beans, cheese, and salad vegetables is more unique to vegetarians in our cohort in Alberta, Canada, or this could be a reflection of the contemporary nature of our data, as studies such as the SWS, NHSII and ALSPAC all described patterns in dietary data collected between 1991 and 2002, while our data were collected between 2009 and 2012. The “tea and coffee” pattern is also dissimilar to others described in women. Whilst dietary patterns that include coffee, tea, and the accompaniments have been described, they often also include other food groups [39,40], and are not as strikingly focused on just these two hot drinks. As with the “beans, cheese and salad” pattern, the novelty of this pattern could be due to geographical differences in the dietary behaviours, or a temporal effect reflective of more modern food habits. 

All, except the “tea and coffee”, dietary patterns had associations with ethnic background with non-Caucasian women being more likely to have a higher “healthy” pattern or “meat and refined carbohydrate” pattern scores and those who were from a Caucasian background more likely to have higher “beans, cheese and salad” pattern scores. Our cohort had a majority of women from a Caucasian background (84%) and so the ethnicity variable was dichotomized to Caucasian and non-Caucasian. In order to explore these further analyses between the sociodemographic characteristics and diet scores were run with the “non-Caucasian” group disaggregated into the individual ethnic categories. This indicated that there were significant differences in the “healthy pattern” score between Caucasians and Chinese (*n* = 59), Native Americans (*n* = 9), and South East Asians (*n* = 52). There were significant differences in the “refined carbohydrate pattern” score between Caucasians and Chinese, Filipinos (*n* = 20), Arabs (*n* = 6), Koreans (*n* = 5), and South East Asians, and there were significant differences in the “cheese, bean and salad pattern” scores between Caucasians and Chinese, Filipinos, and South East Asians. In a sensitivity analysis where these individual groups were included and then excluded we found that significant differences between “Caucasian” and “non-Caucasian” remained with the “healthy” and “bean, cheese and salad” pattern scores, although these associations were attenuated (supplementary Table S4). It is possible that when the smaller ethnicity groups are combined this larger single group provides more power to find a statistically significant association between non-Caucasians and dietary pattern score compared to the reference group (Caucasians). It is challenging to make an inference as to the differences in these dietary pattern scores by ethnicity as the food list in the FFQ may not have been culturally appropriate, thus missing important elements of their habitual diets. Research needs to be repeated in cohorts with greater representation from non-Caucasian ethnicities.

Women who ate a diet more in line with the “healthy” pattern were less likely to develop gestational hypertension in pregnancy; this association was significant after adjustment for pre-pregnancy BMI and other covariates. Other studies which have assessed dietary patterns and hypertensive disorders in pregnancy have reported similar results [19,41,42]. The most prominent dietary pattern assessed during pregnancy in >23,000 women in the Norwegian Mother and Child Cohort Study (MoBa) was one which was characterized by frequent intakes of vegetables, vegetable oils, and rice. In turn they reported that higher scores on this pattern were associated with a lower risk of developing pre-eclampsia [41]. In the Generation R study, a large mother and child cohort study in the Netherlands, 3187 pregnant women had dietary patterns assessed using factor analysis [42]. They described a diet pattern characterized by high intakes of vegetables, vegetable oil, pastas, fish, and legumes, which they termed a Mediterranean pattern. Women who had lower scores on the Mediterranean pattern had higher blood pressure during pregnancy, although this was not significantly associated with the development of gestational hypertension. However, in the Australian Longitudinal Study on Women’s Health, a higher adherence to a Mediterranean pattern prior to pregnancy was shown to be associated with lower risks of developing gestational hypertension in 3582 women [19]. The Mediterranean diet is one rich in vegetables, fruit, pulses, nuts, fish, and olive oil [43]. It has shown to be associated with lower systolic and diastolic blood pressure [44], with the hypothesized mechanisms being that such foods are rich in minerals known to reduce blood pressure, including magnesium, potassium, and calcium. Similarities between the “healthy” pattern in our study and the Mediterranean diet pattern are one possibility for its association with a reduction in the risk of developing gestational hypertension. This highlights the importance of establishing healthy dietary patterns prior to pregnancy, and in between pregnancies, to improve pregnancy health and further indicates that these may be opportune times in the life-course for intervention.

We observed a heightened risk of developing gestational hypertension in those with higher household incomes. This is an unusual finding as income, as other markers of being materially advantaged are more often associated with lower risks of adverse health outcomes [45]. Further analyses showed that the effect was driven by those in the highest group for income (≥$100,000 CAD per year) (data not shown). It is possible that there are lifestyle traits associated with the highest incomes, perhaps including longer working hours or greater stress, that could be driving this observation. We observed a similar association between women with higher incomes and higher intakes of less healthy foods during pregnancy in the same cohort [46]. We also observed that women who were multiparas had lower odds of developing gestational hypertension. This has been reported in other studies assessing the risk factors associated with developing gestational hypertension and pre-eclampsia [47]. One mechanism theorized for this is that there is an immunologic aetiology [48]. The normal immunological response of pregnant women is tolerant to the semi-allogeneic (sharing some but not all genes) foetus, however a defective maternal–foetus immune response may, in part, increase the risk of pregnancy complications, including gestational hypertension and pre-eclampsia. 

We also observed marginally lower odds of developing gestational diabetes in those who reported being more physically active prior to pregnancy; this association was independent of women’s age and pre-pregnancy BMI. This is consistent with other studies which have reported greater physical activity both prior to and during pregnancy is associated with lower risk of gestational diabetes [49,50]. However, we only had 49 women in our cohort who developed gestational diabetes; therefore, the effects may have been more pronounced if we had observed more events. Greater frequency of physical activity prior to and during pregnancy has also been shown previously to reduce the risks of excessive GWG and pre-eclampsia [50]. Therefore, physical activity behaviours prior to pregnancy are also important targets for future interventions to improve health during pregnancy and reduce the risk of pregnancy complications.

### 4.1. Implications

Currently there are clinical guidelines that suggest that women should receive nutrition counselling during pregnancy. Specifically, the IOM implementation of the GWG guidelines [3] and Health Canada’s advice to practitioners [51] is to offer nutrition support to women as early in pregnancy as possible, and to especially target women who are overweight or obese. Whilst our findings did show that those who were obese had lower healthy pattern scores and a greater risk of gestational hypertension, the effects of the healthy pattern on gestational hypertension remained significant following adjustment for BMI, thus supporting the idea that universal nutrition strategies should be developed for all women of childbearing age. Whilst changing dietary behaviour is challenging, it is important to turn attention to ways to support women to establish healthy behaviours prior to pregnancy. This is especially prudent given the evidence that dietary behaviours track from before to during pregnancy [15], and from mother to child [29], thus influencing the dietary behaviours of the next generation. An important next step would be to work with both women of reproductive age and health services to co-develop these strategies so that they are fit for purpose. 

### 4.2. Strengths and Limitations

This was a large prospective study of women’s diets reported prior to pregnancy and objective assessment of the development of complications, including gestational hypertension and diabetes in pregnancy. The APrON study represents the most contemporary birth cohort study of diet and health of mothers during pregnancy and their children in Canada. 

We assessed diet before pregnancy in our cohort of women who were recruited to our study in early pregnancy (median gestational age at recruitment was 17 weeks) who were asked to recall their diet before becoming pregnant on a 142-item FFQ. Whilst this may have exposed the data to issues of recall bias, in a previous study the relative validity of this FFQ in its ability to assess pre-pregnancy diet was assessed [23]. The FFQ was administered to two groups of individuals a sub-cohort of 91 pregnant women randomly selected from the main APrON cohort and a control group of 101 non-pregnant women. Women who were pregnant were asked to report their diet in the 12 months before becoming pregnant and the non-pregnant women were asked to report their diet in the previous 12 months. Independent *t*-tests of the mean intakes of energy, macronutrients, iron, folate, and vitamin B6, B12, or D between these two sub-cohorts showed no differences at the group level. Furthermore in the Southampton Women’s Survey women reported their diet using an FFQ prior to pregnancy and again in early pregnancy and dietary patterns analysis was performed. This showed that there was high correlation (*r* = 0.71) between the dietary pattern scores assessed prospectively at these two time points [15]. Therefore an FFQ completed in early pregnancy was deemed appropriate for use of the collection of pre-pregnancy dietary data.

Pre-pregnancy weight data was self-reported by the participants, which may have caused some women to be misclassified in their BMI categories. However analyses carried out as part of a previous study with this cohort found that in a sub group of 528 women who self-reported pre-pregnancy weight and then had weight measured in their first trimester (when weight gain is limited), >90% of women were correctly classified into their respective BMI categories [26]. The women in the APrON cohort tended to be highly educated, with high household incomes, and from white ethnic backgrounds, which is a similar participant profile to that reported in the Australian Longitudinal Study on Women’s Health [17] and Generation R [42]. Whilst this is not representative of the population of women of childbearing age in Canada as a whole, comparisons with other studies which have wider variation in the demographics of their cohort indicate that our findings are likely generalizable to the wider population. For instance, participants in the MoBa study were more representative of the wider population, with only 20.5% having university-level education and >28% being overweight or obese [41]. They reported finding a similar “healthy” dietary pattern to the one in our study, which was associated with a decreased risk of pre-eclampsia. Therefore, the associations reported in the somewhat restricted APrON sample are likely to be robust effects which may be underestimated compared to the general population. Women experiencing lower incomes or who live in more vulnerable situations due to lower educational attainment may require more support to achieve the ‘healthy’ dietary pattern described here. Lastly, subjective decisions were made during the application and interpretation of dietary patterns analysis using PCA. The study team was multidisciplinary, consisting of nutrition experts and epidemiologists to reduce challenges with the analysis and its interpretation.

## 5. Conclusions

This study adds to the growing evidence that preconception diet and lifestyle is crucial for the optimal health of the mother, and possibly her offspring, and needs to be the focus for future health interventions. The next steps are to work in multidisciplinary teams, with representation from women of childbearing age and healthcare providers, to co-develop initiatives which support women to establish better eating patterns for their own health and that of the next generation.

## Figures and Tables

**Table 1:** Participant characteristics. BMI: body mass index.

Characteristics (*n* = 1545)	
Age in years (mean (SD))	31.4 (4.2)
Education (*n* (%))	
- Below university level	420 (28)
- University level	1102 (72)
Household yearly income (*n* (%))	
- <$100,000	634 (42)
- ≥$100,000	883 (58)
Marital status (*n* (%))	
- Single, divorced, widowed	45 (3)
- Married/cohabiting	1483 (97)
Parity (*n* (%))	
- Nulliparous	861 (57)
- Multiparous	662 (43)
Ethnicity (*n* (%))	
- White	1288 (84)
- Not white	237 (16)
Smoked during pregnancy (*n* (%))	12 (1)
Pre-pregnancy BMI (*n* (%))	
- Underweight	48 (3)
- Normal weight	913 (65)
- Overweight	295 (21)
- Obese	154 (11)
Gestational weight gain guidelines (*n* (%))	
Below	247 (18)
Within	465 (34)
Above	666 (48)
Developed gestational hypertension (*n* (%))	93(6)
Developed gestational diabetes (*n* (%))	49 (3)

**Table 2:** Dietary patterns derived from principal component analysis and their corresponding coefficients.

Food Groups	Healthy Pattern	Meat and Refined Carbohydrate Pattern	Beans, Cheese and Salad Pattern	Tea and Coffee Pattern
**Green vegetables**	**0.36**	−0.03	0.01	−0.04
**Orange vegetables**	**0.30**	−0.07	0.07	−0.05
**Other vegetables**	**0.36**	0.07	−0.06	−0.01
**Salad vegetables**	0.06	−0.07	**0.46**	−0.07
**Beans and pulses**	−0.005	−0.05	**0.47**	−0.08
**Vegetarian food**	0.14	−0.14	−0.007	−0.02
**Tomatoes**	**0.20**	0.09	0.09	−0.01
**Boiled potatoes**	0.005	**0.20**	0.005	−0.01
**Fries**	−0.05	**0.30**	−0.007	−0.02
**Chips**	0.06	0.06	0.12	−0.01
**Fruit juice**	0.06	0.07	−0.03	0.03
**Fruit**	**0.33**	−0.10	0.07	0.009
**Dried fruit**	0.19	−0.13	0.008	0.11
**Nuts and seeds**	0.10	−0.02	0.15	0.04
**Confectionery**	−0.04	0.15	0.15	0.06
**Sweet spreads**	0.06	0.07	0.06	0.05
**Added sugar**	−0.03	0.08	−0.03	**0.27**
**White bread**	−0.01	**0.26**	−0.05	0.001
**Whole-meal bread**	0.05	0.16	0.10	0.02
**Brown rice and pasta**	**0.22**	−0.05	0.08	0.02
**White rice and pasta**	**0.20**	0.17	−0.14	−0.002
**Breakfast cereals**	0.07	−0.08	0.11	0.07
**Crackers**	0.04	0.006	0.11	0.02
**Pizza**	0.07	0.11	0.14	−0.04
**Perogies and dumplings**	0.06	0.19	−0.08	−0.007
**Pancakes and waffles**	0.03	0.07	0.02	0.04
**Cakes and biscuits**	−0.009	0.14	0.13	0.03
**Desserts**	0.001	0.17	0.10	0.01
**Full-fat milk**	0.05	0.004	−0.14	**0.25**
**Reduced-fat milk**	0.01	−0.04	0.04	**0.46**
**Cream**	−0.08	0.17	0.11	**0.27**
**Cheese**	−0.09	0.04	**0.46**	−0.05
**Yoghurt**	0.13	0.02	0.08	0.04
**Protein bars**	−0.008	−0.02	0.03	−0.03
**Full-fat spread**	0.10	0.08	0.01	−0.05
**Reduced-fat spread**	0.05	0.14	0.05	−0.02
**Oils**	**0.28**	0.08	−0.02	0.01
**Eggs**	0.19	0.07	−0.03	−0.01
**Chicken and turkey**	0.07	0.17	−0.003	−0.005
**Red meat**	0.06	**0.37**	−0.04	0.01
**Processed meat**	−0.06	**0.34**	0.03	0.0008
**Fish and shell fish**	0.22	0.14	−0.08	0.02
**High-energy soft drinks**	−0.10	0.18	−0.02	0.01
**Diet soft-drinks**	−0.07	0.07	0.08	−0.01
**Tea**	0.05	−0.08	−0.02	**0.38**
**Decaf tea**	0.06	−0.12	0.02	**0.38**
**Coffee**	−0.08	0.04	0.06	**0.32**
**Decaf coffee**	−0.02	−0.02	0.006	**0.22**
**Wine**	−0.07	0.007	0.18	0.14
**Beer and liquor**	−0.09	−0.02	0.03	−0.03
**Miscellaneous**	0.06	0.11	0.008	0.13

Text in bold highlights food groups which have a coefficient > 0.2 and characterize the pattern.

**Table 3:** Beta coefficients (95% confidence intervals) from multivariate linear regressions of socio-demographic characteristics associated with diet pattern *z*-scores.

Characteristic	Healthy Pattern	Meat and Refined Carbohydrate Pattern	Beans, Cheese and Salad Pattern	Tea and Coffee Pattern
**Age in years**	0.005	−0.01	NS	0.02 *
**Education**				
- Less than university ^†^	-	-	-	-
- University level	0.27 ***	−0.13 **	0.09	0.03
**Income**				
<$100,000 CAD ^†^	-	-	-	-
≥$100,000 CAD	−0.08	−0.05	0.16*	NS
**Ethnicity**				
- Caucasian ^†^	-	-	-	-
- Non-Caucasian	0.52 ***	0.38 ***	−0.43 ***	−0.13
**Parity**				
- Nulliparous ^†^	-	-	-	-
- Multiparous	NS	NS	−0.06	NS
**BMI**				
- Underweight	−0.03	NS	NS	NS
- Normal weight ^†^	-	-	-	-
- Overweight	0.02	NS	NS	NS
- Obese	−0.23 *	NS	NS	NS
**Smoking in pregnancy**	NS	NS	NS	NS
**Physical activity index**	0.04 *	NS	0.05 *	NS

NS = Non-significant (*p* > 0.2) in univariate analysis * *p* < 0.05; ** *p* < 0.01; *** *p* < 0.001. ^†^ Reference category. All regression analyses were also adjusted for total energy intake.

**Table 4:** Associations with the development of gestational hypertension.

Variable	Odds Ratio	95% Confidence Intervals	*p*-Value
Pre-pregnancy BMI ^†^	2.7	1.9, 3.8	<0.001
Income ^††^	1.6	1.0, 2.5	0.03
Parity	0.5	0.2, 0.9	0.02
Healthy eating pattern score	0.6	0.4, 0.9	0.01

Forward step-wise logistic regression model. Factors which had a univariate association with gestational hypertension of *p* < 0.2 were included in the final model: Maternal age, total gestational weight gain, total physical activity index and total daily energy intake (kcals). ^†^ Pre-pregnancy BMI in three groups; 1 = normal weight, 2 = overweight, 3 = obese. ^††^ Income in five ordered groups: “<$20,000 CAD”, “$20,000–$39,999 CAD”, “$40,000–$59,999 CAD”, “$60,000–$79,999 CAD”, “$80,000–$99,999 CAD”, and “≥$100,000 CAD”.

## Supplementary Materials

The following are available online at http://www.mdpi.com/2072-6643/10/7/914/s1, Table S1: Food groups and the FFQ items included in them, Table S2: Associations with gestational diabetes, Table S3: Associations with gestational weight gain (GWG) in excess of the IOM guidelines (compared with GWG within the IOM guidelines), Table S4: Sensitivity analysis showing relationship between ethnicity and dietary pattern scores with and without the individual ethnic groups in the non-Caucasian category associated with the patterns.
