# Supplementary material for: Dietary Patterns Prior to Pregnancy and Associations with Pregnancy Complications
Source: Nutrients. 2018 Jul 17;10(7):914. doi: 10.3390/nu10070914 (PMC6073508; doi:10.3390/nu10070914)
Supplement: Supplementary file 1 [file nutrients-10-00914-s001.pdf]

Supplementary table 1: Food groups and the FFQ items included in them

**Food group**	**Included items from the FFQ**
White bread	White bread (including bagels, pitta, English muffins, croissants etc.)
Wholemeal bread	Wholemeal bread (including wholemeal versions of any bread product)
Cereals	'Bran' breakfast cereals/cereal bars Non-bran breakfast cereals and cereal bars Oatmeal, Cream of Wheat, Other cooked cereals
Crackers	Crackers, Wholemeal crackers, Rice cakes
White rice and pasta	White Pasta, White Rice, Mixed pasta dishes
Cakes, Muffins, and Cookies	Cakes, Muffins, Pastries, Chocolate cookies, Banana Breads, Sweet Muffins, Dessert Breads, Other fruit breads, Other cookies
Puddings and desserts	Fruit based puddings, Milk based puddings, Other desserts, Pies, Ice cream, frozen yogurt
Full Fat Milk	Full Fat Liquid milk (>3.2% M.F.), Homo Milk
Reduced Fat Milk	1% Milk, 2% Milk, Powdered Milk, Skim Milk, Soy Milk
Cream	Sour Cream, Cream/half & half
Cheese and cheese sauce	Cheese, Imitation cheese, Cheese Sauce
Yoghurt and Cottage Cheese	Yoghurt, Cottage Cheese
Eggs and egg dishes	Eggs, Omelets, Egg dishes
Full Fat spreads	Full-fat spread (>69g fat/100g), Mayonnaise, Butter, Lard
Reduced Sat. Fat Spreads	Margarine (including low-fat), Reduced-fat spread (<69g fat/100g), Reduced-fat artificial creamer
Oils	Canola oil, Olive oil, Vegetable oil, Regular artificial Creamer
Poultry	Chicken and turkey (not processed)
Red meat	Pork, Lamb, Beef, Minced meat dishes
Processed meat	Bacon and gammon, Meat pies, Sausage, Ham and canned meats Luncheon meats, Canned chili
Fish and shell-fish	White fish, Fish pie, Oily fish, Shell-fish
Salad Vegetables excluding tomatoes	Salad, Coleslaw, Cucumber, Arugula Belgium endive/chicory, Green pepper & watercress, Avocado, Olives

Green vegetables	Spinach, Kale and collards, Sprouts and broccoli, Cabbage and cauliflower, Peas, Zucchini, marrow & leeks, Celery, Artichokes, Asparagus, Brussels sprouts
Dark yellow/Orange vegetables	Carrots, Rutabaga, Yams, Sweet potatoes, Pumpkin, Butternut squash, Winter squash
Other vegetables	Sweet corn, Turnip, Parsnips, Onion, Mushrooms, Mixed fresh or frozen vegetables, Seaweed
Beans and pulses	Beans, chickpeas, lentils
Boiled potatoes	Boiled potatoes
Fries	Fries, Roast potatoes
Chips and Popcorn	Chips, fried snacks, Popcorn, Pretzels
100% Fruit and Vegetable Juices	Orange juice, Grapefruit juice, Other fruit juices, Smoothies, Vegetable Juices, Tomato Juice
Whole fruit	Apples and pears, Banana, Peaches, plums, apricots, cherries and grapes, Strawberry and raspberry Melon, Pineapple, Mango, Kiwi, Oranges, Grapefruits
Nuts and seeds	Nuts and seeds
Dried fruit	Dried fruit
Confectionery	Chocolate, Candy, Other confectionery
Sweet spreads	Sweet spreads, Jam
Added sugar	Added sugar (teaspoons), Coffee syrups, Pancake syrup, Maple syrup
High-energy soft drinks	High sugar drinks, Fizzy drinks, Coke, Energy drinks
Low energy drinks	Diet sodas, Reduced sugar drinks, Flavoured waters
Tea	Tea
Coffee	Coffee
Decaffeinated tea	Decaffeinated tea and herbal tea
Decaffeinated coffee	Decaffeinated coffee
Wine	Wine and fortified wine
Nutrition bars and drinks	Protein and energy bars, Protein drinks
Pancakes and waffles	Pancakes, French Toast, Waffles, Crepes
Tomatoes	Tomatoes, Tomato paste, Canned tomatoes, Ketchup, Salsa

Pizza and related mixed dishes	Pizza, Empanadas, Tacos, Burritos, Fajitas
Meat Substitutes and vegetarian foods	Soy Meat-substitutes, Tofu, Vegetarian Foods
Beer and hard liquor	Beer, Hard liquor (vodka, Scotch, etc.)
Miscellaneous	Gravy, Salad dressings, Artificial Sweetener
Dumplings and Perogies	Dumplings, Perogies

Supplement Table 2: Associations with gestational diabetes

Variable	Odds ratio	95% confidence intervals	P Value
Pre-pregnancy BMI†	2.3	1.4, 3.6	0.001
Age (years)	1.16	1.1, 1.3	0.001
Total physical index	0.66	0.5, 0.9	0.007

Forward step-wise logistic regression model. Factors which had a univariate association with gestational diabetes of p<0.2 were included in the final model: Education (in 2 groups) Ethnicity (in 2 groups), total gestational weight gain, and total daily energy intake (kcals).

†Pre-pregnancy BMI in 3 groups; 1= normal weight, 2 = overweight, 3 = obese.

Supplement Table3: Associations with gestational weight gain (GWG) in excess of the IOM guidelines (compared with GWG within the IOM guidelines)

Variable	Odds ratio	95% confidence intervals	P Value
Pre-pregnancy BMI†	2.2	1.8, 2.7	<0.001
Education††	0.8	0.6, 1.1	0.16
Age (years)	1.0	0.9, 1.0	0.16
Tea & Coffee pattern score	1.1	0.98, 1.3	0.07

Forward step-wise logistic regression model. Factors which had a univariate association with gestational weight gain (in excess versus within the GWG guidelines) of p<0.2 were included in the final model: Household income (in 2 groups) and total daily energy intake (kcals).

†Pre-pregnancy BMI in 3 groups; 1= normal weight, 2 = overweight, 3 = obese.

†† Education in 2 groups; 1= >university level, 2= ≥university level

Supplement Table 4: Sensitivity analysis showing relationship between ethnicity and dietary pattern scores with and without the individual ethnic groups in the non-Caucasian category associated with the patterns.

Characteristic	Healthy pattern	Refined carbohydrate pattern	Beans cheese and salad pattern
Ethnicity			
- Caucasian (reference group)	--	--	--
- Non-Caucasian	0.52***	0.38***	-0.43***
Ethnicity			
- Caucasian (reference group)	--	--	--
- Non-Caucasian†	0.22*	0.21	-0.29*

Linear regression models with pattern scores as the outcome variable. Models also adjusted for participant age, education, income, parity, pre-pregnancy BMI, physical activity score and total daily Kcal intake.
* P <0.05; ** P <0.01; *** P <0.001

†Excluding Chinese, Native American and South East Asian groups in analysis with healthy pattern; Excluding Chinese, Filipino, Korean, Arab and South East Asian groups in analysis with refined carbohydrate pattern; Excluding Chinese, Filipino and South East Asian groups in analysis with beans, cheese and salad pattern
